# Dual Viscosity Mixture Vehicle for Intratympanic Dexamethasone Delivery Can Block Ototoxic Hearing Loss

**DOI:** 10.3389/fphar.2021.701002

**Published:** 2021-10-28

**Authors:** Hui Li, Myung-Whan Suh, Seung Ha Oh

**Affiliations:** ^1^ Department of Otorhinolaryngology-Head and Neck Surgery, Seoul National University College of Medicine, Seoul, South Korea; ^2^ Department of Otorhinolaryngology-Head and Neck Surgery, Seoul National University Hospital, Seoul, South Korea

**Keywords:** high molecular weight, hyaluronic acid, auditory brainstem-evoked responses, hearing loss, inner ear

## Abstract

Clinically there is no effective method to prevent drug induced hearing loss in patients undergoing chemotherapy and anti-tuberculosis therapy. In this study, we developed an intratympanic (IT) local drug delivery vehicle featuring hyaluronic acid-based dual viscosity mixture encapsulation of dexamethasone (D), named dual-vehicle + D, and assessed its protective effect in ototoxic hearing loss. We assessed the residence time, biocompatibility, and treatment outcome of the novel vehicle compared with the current standard of care vehicle (saline) and control conditions. The hearing threshold and hair cell count were significantly better in the dual-vehicle + D group compared to the other two groups. The final hearing benefit in the dual-vehicle group was approximately 25–35 dB, which is significant from a clinical point of view. Morphologic evaluation of the cochlear hair cells also supported this finding. Due to the high viscosity and adhesive property of the vehicle, the residence time of the vehicle was 49 days in the dual-vehicle + D group, whereas it was less than 24 h in the saline + D group. There was no sign of inflammation or infection in all the animals. From this study we were able to confirm that dual viscosity mixture vehicle for IT D delivery can effectively block ototoxic hearing loss.

## Introduction

Corticosteroid is one of the most widely used medications for various inner ear disorders. However, delivering a sufficient amount of steroid over a prolonged duration into the inner ear is not easy. Meniere’s disease, idiopathic sudden sensorineural hearing loss (SNHL), chronic progressive SNHL, noise-induced hearing loss, and immune-mediated hearing loss have been shown to respond to steroids (Hughes et al., 1984; Harris, 1991; Gilles et al., 2014; Chen et al., 2015; Kil et al., 2017). Based on level 2 evidence, the American Academy of Otolaryngology–Head and Neck Surgery has recommended steroid administration as salvage therapy for sudden SNHL (Chandrasekhar et al., 2019). The European Academy of Otology and Neurotology has recommended the use of steroids for the treatment of Meniere’s disease (Magnan et al., 2018). However, both systemic and local intratympanic (IT) administration of steroids are associated with considerable challenges. For instance, systemic steroid administration can deliver only a small amount of steroid into the perilymph, due to the blood labyrinth barrier (Bowe and Jacob, 2010; Bird et al., 2011). The adverse effects of systemic steroid use, such as abdominal discomfort, rashes, hot flushes, and toxic hepatitis, should also be considered (Min et al., 2012). Compared to systemic steroids, IT steroid administration can deliver 33–126-fold higher concentrations of steroid into the inner ear and is free of systemic adverse effects (Bird et al., 2007; Wang et al., 2011). However, the duration of effective drug delivery is less than several hours (Wang et al., 2009). Due to this short-term effect, multiple IT injections are needed in most patients. In some cases, repeated puncture of the tympanic membrane (TM) may lead to infection and permanent TM perforation (Syed et al., 2015).

IT drug delivery vehicles that reside in the middle ear and release the drug over a prolonged period can be a practical solution. It is presumed that preventing drug/vehicle drainage through the Eustachian tube is one mechanism of prolonging the IT drug delivery effect (Wang et al., 2009; Lambert et al., 2012; El Kechai et al., 2015). Several vehicles have been developed based on this idea. Poloxamer 407 (Wang et al., 2009), gelatin (Inaoka et al., 2009), chitosan (Paulson et al., 2008; Saber et al., 2010), and collagen (Endo et al., 2005) are good candidates. We have also recently reported sustained delivery from the use of methoxy polyethylene glycol-b-polycaprolactone block copolymer (Park et al., 2020) and click-crosslinking hyaluronic acid (HA) (Ju et al., 2020). These new IT drug delivery vehicles confer some advantages over the current standard-of-care vehicle (physiologic saline). However, the therapeutic effects and/or biocompatibility of most vehicles are not sufficient for clinical use.

In this study, we focused on a HA-based dual viscosity mixture as a novel vehicle. HA is a natural lubricant and biopolymer material with high water solubility, biocompatibility, biodegradability, and non-toxicity (Rice, 1998). HA has been approved for treating osteoarthritis pain and for use in eye surgeries by the United States Food and Drug Administration (Fallacara et al., 2018). In the ear, we found no cases of inflammation or infection in our former study after IT HA injection (Park et al., 2020). Thus, HA appears to be a safe, biocompatible material for use in the human body, including the ear. Prolonged sustainability is another advantage of HA. It has been reported that HA can last in the middle ear for up to 30 days (El Kechai et al., 2016), whereas poloxamer 407 lasts for 10 days (Wang et al., 2009; Wang et al., 2011), chitosan glycerophosphate for 5 days (Paulson et al., 2008), and thiol-modified HA/gelatin hydrogel for 3 days (Borden et al., 2011). To further potentiate the sustained-release property of HA, we produced a dual viscosity mixture with HA. Dual viscosity mixture HA are colloidal dispersions with two immiscible aqueous phases that are in thermodynamic equilibrium, often referred to as an aqueous two-phase system. Because the interfacial tension is very low, beyond the critical point, the two immiscible water-phase transitions disappear (Esquena, 2016). The outer high-viscosity HA (with high surface tension) works as an adhesive scaffold (Girish and Kemparaju, 2007), as it is resistant to being drained through the Eustachian tube and adheres to the round window (RW) and oval window (OW). Meanwhile, the inner low-viscosity HA works as a drug-loaded depot. The use of dual viscosity mixture vehicle can significantly reduce dosage frequency and improve drug efficacy via a single injection (Peters et al., 2017).

Dexamethasone-loaded, low-molecular-weight HA were prepared and mixed with high-molecular-weight HA, resulting in a dual viscosity mixture vehicle (dual vehicle). Our hypothesis was that the multiphase dual vehicle would allow sustained release of the steroid with no adverse effects, resulting in superior functional outcomes. We assessed 1) whether the dual vehicle can last in the middle ear for up to several weeks, 2) whether our treatment has a clinically significant preventive effect on ototoxic hearing loss, and 3) the safety and biocompatibility of the novel vehicle.

## Materials and Methods

### Experimental Animals and Preparation of the Drug/Vehicle

This study was approved by the Animal Research Committee of Seoul National University Hospital, and all animal care was supervised by the Institutional Animal Care and Use Committee Institute (IACUC16-0243-01A02). All animals were maintained on a 12:12 h light dark cycle and the average experimental time was 1.35 ± 2.67 h/day (for 49 days). Twenty-three (46 ears) male Sprague Dawley rats (6 weeks old; 165–245 g) were used for this study. The ears were categorized into three groups, according to the treatment strategy. The first two groups were treated with the same concentration (12 mg/ml) of IT dexamethasone phosphate (D); however, the vehicles for delivering dexamethasone differed between the two groups. Dexamethasone was mixed inside the low molecular weight HA in the dual-vehicle + D group (*n* = 20), whereas in the saline + D group (*n* = 16), the vehicle was physiologic saline. The control group (*n* = 10) did not receive any treatment before or after induction of ototoxic hearing loss. For each animal, the left and right ears were allocated in such a way that individual animal-associated variability was minimized.

**FIGURE 1:**
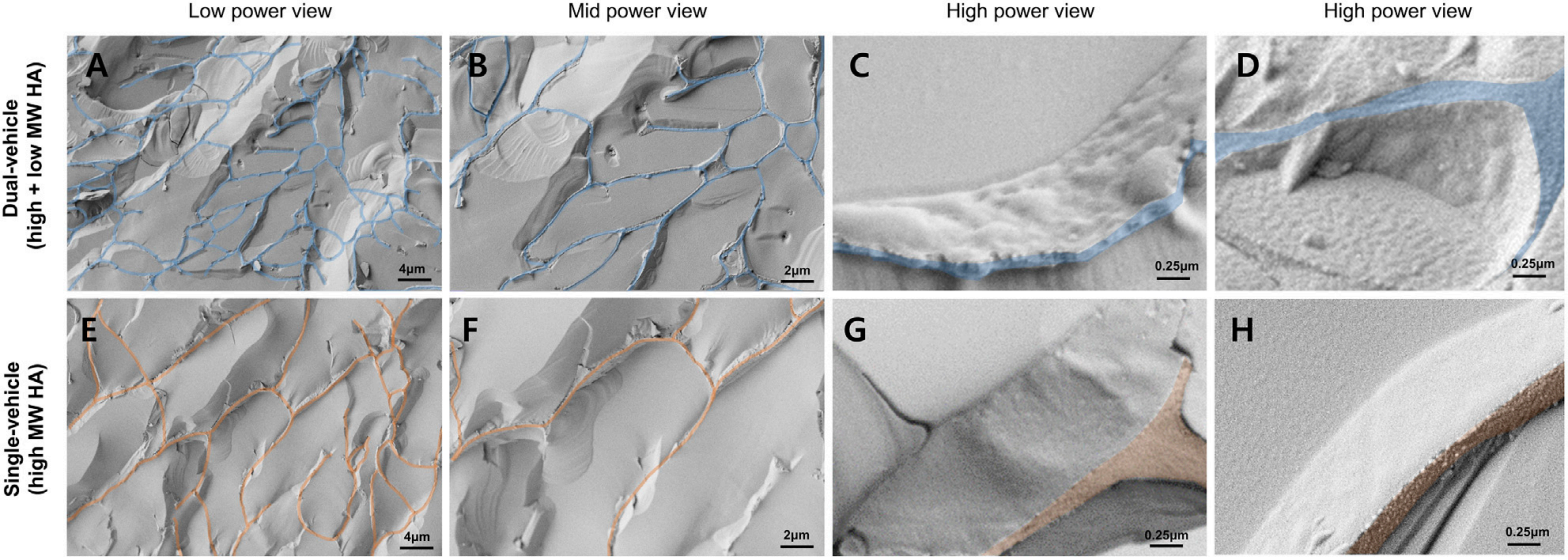
Cryo-scanning electron microscopy of the dual-vehicle. The slab-like structures are the interconnected fibrous hyaluronic acid (HA) network. The cutting planes of the fibrous HA network were colored coded in blue and red. The glass-like structure inside the HA network are the frozen aqueous media. Compared to the single-vehicle [only high molecular weight (MW) hyaluronic acid, **(E,F)**], the average pore dimension was smaller in the dual-vehicle **(A,B)**. Also, freezing of the two different phases of HA made the surface texture of the fibrous HA network more granular **(C,D)**. Surface texture of the single-vehicle HA network was very smooth **(G,H)**.

The specific composition and preparation process of the drug/vehicle are reported elsewhere (Kang, 2020). In brief, dexamethasone disodium phosphate (water-soluble salt form) was prepared in 10 mM phosphate buffer at a concentration of 1.2% (w/v). Low-molecular-weight HA (10–100 kDa, MNH Bio Co. Ltd. Hwaseong-si, Korea) was completely dissolved in the solution at a concentration of 1.0% (w/v) to coat the drug. Dexamethasone mixed in low-molecular-weight HA was prepared. High-molecular-weight HA (5,000–10,000 kDa, MNH Bio Co. Ltd. Hwaseong-si, Korea) was dissolved in the dexamethasone-low-molecular-weight HA solution by vigorous mixing until a concentration of 2.0% (w/v) was reached (Figure 1). Hyaluronic acid was filtered with a 0.2 μm cellulose acetate membrane to prevent bacterial and mold contamination. As for the dexamethasone sodium phosphate, the total aerobic microbial count was less than 103 cfu/g, total combined mold and yeast count was less than 102 cfu/g, and bacterial endotoxins were less than 0.25 EU/mg. Loading the drug in the vehicle was performed in a sterilized container and environment.

### IT Drug Delivery

The rats were anesthetized with zoletil and xylazine. IT dexamethasone (ITD) administration was performed under a surgical microscope (OPMI Pico, Carl-Zeiss, Oberkochen, Germany). An Angiocath Plus 24-gauge needle (BD, Sandy, UT, United States) was connected to a 1-ml syringe (Kovax-Syringe 1 ml, Korea Vaccine Co., Seoul, Korea) with a mini-extension tube (Mini-Volume Line, Insung Medical, Seoul, Korea). An air vent was first made in the anterior superior quadrant of the TM. The syringe was inserted carefully at a low speed (∼60 µL/10 s). The injection was stopped when the drug/vehicle completely filled the middle ear (bulla) or the drug/vehicle leaked out through the air vent. The volume of the drug/vehicle injected into the middle ear cavity was similar (40–60 μL) between the dual-vehicle + D group and saline + D group. We achieved good or fair injection quality in >95% of the cases; only these ears were included in the study. After injecting the drug/vehicle into one ear, the other ear was also immediately injected with a different drug/vehicle; the time interval between the first and second injections was <5 min. To prevent position effects, the animals were placed in a straight prone position, without leaning toward the left or right side, until the experiment was concluded. The drug/vehicle that was injected, and the order in which the drug/vehicle was injected, were randomized.

### Induction of Ototoxic Hearing Loss

To induce ototoxic hearing loss, intravenous injection of ototoxic drugs was administered over two consecutive days. After the rats were anesthetized, cisplatin (2 mg/kg, 0.5 mg/ml) and gentamicin (120 mg/kg, 40 mg/ml) solutions were slowly (0.2 ml/2 min) injected through the vein at the lateral side of the tail using an Angiocath Plus 24-gauge needle. Five minutes after flushing the tube with normal saline (0.3 ml), furosemide (90 mg/kg, 10 mg/ml) was slowly (0.2 ml/2 min) injected. Ototoxic hearing loss was induced on the third and fourth days after IT drug delivery. The last day of ototoxic hearing loss induction was considered post-hearing loss day (PHD) 0.

### TM Endoscopy and Micro-Computed Tomography

A 2.7-mm-diameter endoscope (GD-060, Chammed, Gunpo, Korea) was connected to a smartphone (iPhone 4, Apple Inc., Cupertino, CA, United States) to photograph the external auditory canal and TM of the rats. We observed whether there was inflammation, swelling, congestion, perforation, or other adverse effects. The surface integrity, healing of the perforation, and transparency of the TM were also assessed. To evaluate the residual amount of drug/vehicle in the middle ear, a micro-computed tomography (CT) system (NFR Polaris-G90; Nanofocusray Co., Ltd., Jeonju, Korea) was used. TM endoscopy and micro-CT measurements were performed before and at 1 h after IT drug delivery and on day 1, 5, 8, 12, 16, 25, 34, and 49. Five micro-CT sections that covered the entire middle ear were analyzed. The area of the drug/vehicle in each section was measured and stacked to produce three-dimensional volume data.

### Assessing the Hearing Threshold Based on the Auditory Brainstem Response

Using the Smart EP system (Intelligent Hearing Systems, Miami, FL, United States), we evaluated hearing outcomes. In a sound-proof chamber, auditory brainstem response (ABR) threshold tests were conducted at 8, 16, and 32 kHz. Prior to ABR measurements, the animals were anesthetized, as mentioned above. Subdermal needle electrodes were inserted at the vertex (active electrode) and behind the ipsilateral ear (reference electrode) and contralateral ear (ground electrode). The speaker was aligned with the external auditory canal, and the earphone tube was inserted gently into the ear canal. Hearing thresholds were determined by evaluating the lowest stimulus level that produced clear III/V and SN10 (a slow, negative wave) waves. ABR testing started at 90 dB SPL, with a 5-dB decrease each time. ABRs were evaluated at the same time points as with TM endoscopy and micro-CT measurements.

### Organ of Corti Surface Preparation and Hair Cell Count

On the last day of the experiment (PHD 45), the animals were anesthetized, and the cochleae were harvested, perfused with 4% paraformaldehyde in phosphate-buffered solution (PBS), and further fixed with 4% paraformaldehyde at 4°C for 24 h. Organ of Corti surface preparation was performed under a stereoscopic microscope (SZX7, Olympus, Tokyo, Japan). The cochlea was stained with phalloidin (Alexa Fluor 546, Life Technologies, Oregon, United States) and rinsed for 5 min three times with PBS. Hair cells were observed by z-stacking with a confocal microscope (Leica TCS SP8, Leica Microsystems, Wetzlar, Germany). Three rows of outer hair cells were counted within a length of 200 μm in each turn. Two separate samples for each turn were used for measurements. When the hair cells were empty or absent on photomicrographs, the hair cells were considered non-living.

### Middle Ear Histology

On the last day of the experiment, the middle ear was harvested, perfused with 4% paraformaldehyde and PBS, and further fixed with 4% paraformaldehyde for 12 h at room temperature. After decalcification with 0.1 M ethylenediaminetetraacetic acid (pH 7.4) for 3 weeks, the bullae were embedded in paraffin wax, cut into 5-μm-thick sections, and stained with hematoxylin and eosin. The location of the TM was identified in a consistent manner by locating the malleus head and its fibrous connection to the TM. The mucosa at the base of the bulla (BB) was evaluated. Using a light microscope (CX31, Olympus, Tokyo, Japan), the thicknesses of the TM and BB mucosa were measured with DP2-BSW software (Olympus).

### Cryo-Scanning Electron Microscopy

Dual vehicle and single vehicle (only high molecular weight HA) were cryoimmobilized by plunge freezing into a liquid nitrogen bath at −196°C. Frozen samples were fractured and coated with a platinum. After transfer to the SEM chamber, samples were examined at 1.5 kV under high vacuum on the cryo-SEM stage (Crossbeam 550, Carl Zeiss, Oberkochen, Germany).

### Statistical Analysis

All statistical analyses were performed using SPSS software (version 25.0; SPSS Inc., IBM Corp., Armonk, NY, United States). Continuous variables are expressed as the means ± standard deviations in the figures. The Mann–Whitney U-test was used to compare the outcomes in each group. P-values < 0.05 were considered to denote statistical significance.

## Results

### TM Endoscopy and Micro-CT

**FIGURE 2:**
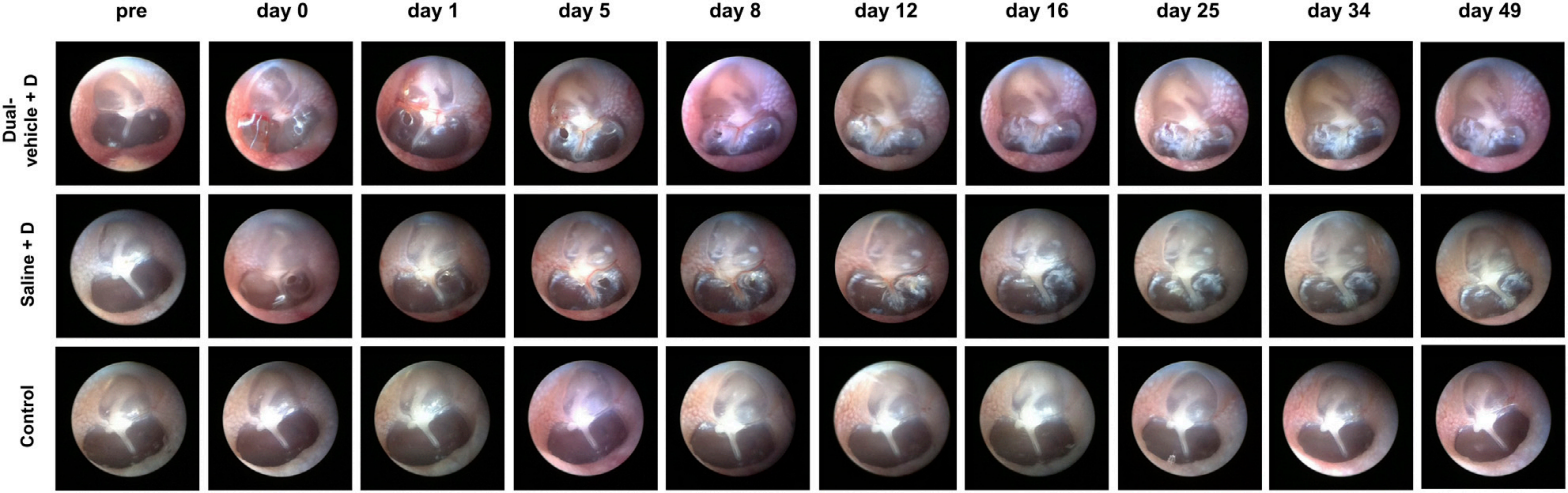
Tympanic membrane (TM) endoscopy after intratympanic (IT) drug delivery. Two small perforations were made during IT drug delivery. The perforations healed well in all ears after 18.4 ± 7.3 and 13.2 ± 7.8 days in the dual-vehicle + D and saline + D groups, respectively. There were no signs of inflammation or infection in any of the ears. No IT intervention was performed in the control group.

The TM perforation made during the IT drug delivery healed well in all animals within 16 days; the results are shown in Figure 2. There was no ear that ended up with a permanent perforation. The incidence of inflammation or infection was 0.0% in all three groups (dual-vehicle + D, saline + D, and control groups). Closure of the perforation was observed at 18.4 ± 7.3 days after IT drug delivery in the dual-vehicle + D group and at 13.2 ± 7.8 days in the saline + D group. The time required for the perforation to heal was also similar between these two groups. In the control group, there was no perforation in the ears, as IT intervention was not performed.

**FIGURE 3:**
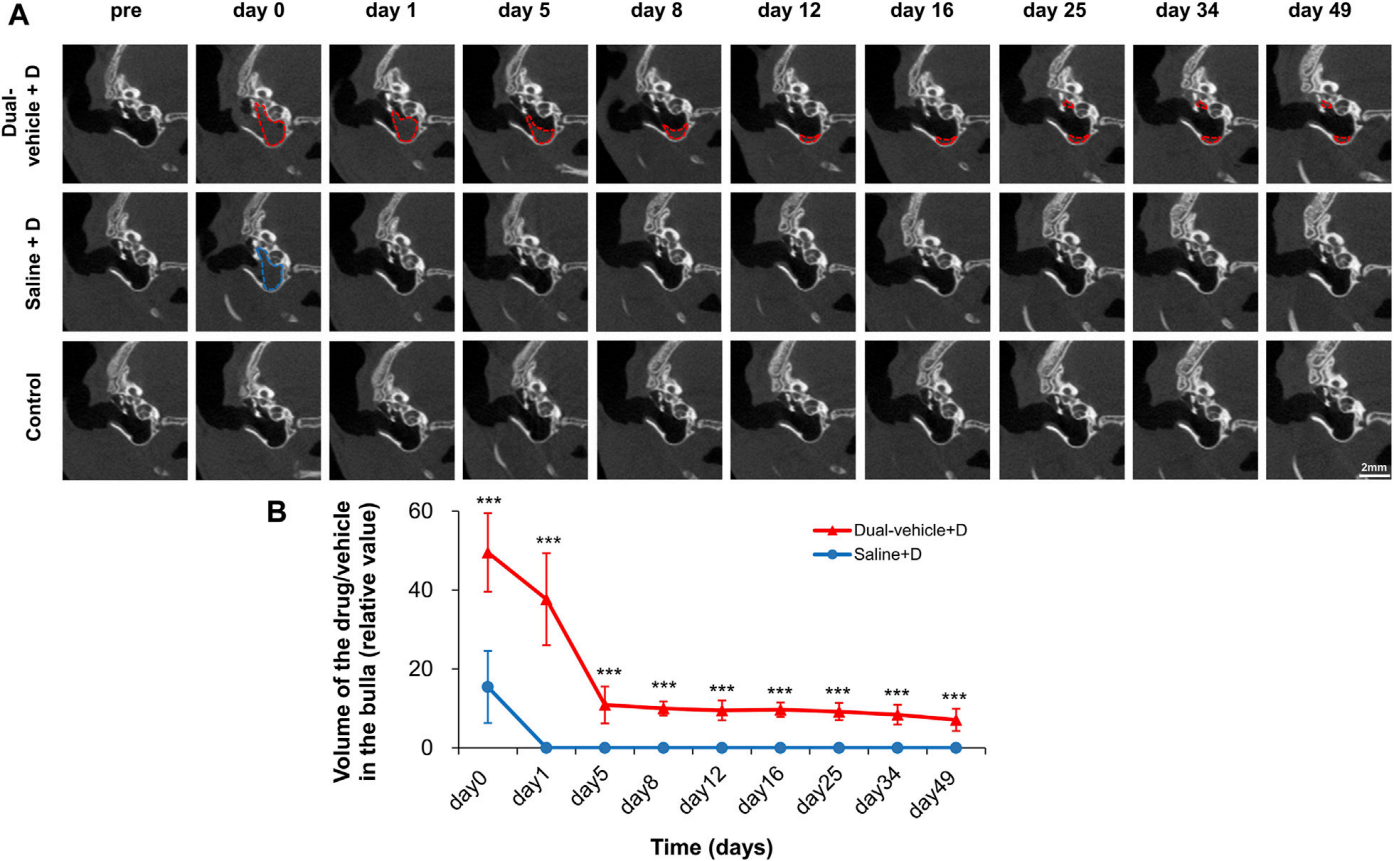
Amount of drug/vehicle remaining in the middle ear after IT drug delivery. The duration of drug/vehicle residence in the middle ear was evaluated using micro-computed tomography (CT). In the dual-vehicle + D group, the middle ear was filled with drug/vehicle on day 0. The volume rapidly decreased within 5 days, but there was still a visible amount of drug/vehicle (indicated by red and blue dotted lines) at up to 49 days **(A)**. In the saline + D group, the amount of drug/vehicle visible decreased much faster from the time of the first micro-CT; after 24 h, there was little evidence of any remaining drug/vehicle **(B)**. *p < 0.05, **p < 0.01, ***p < 0.001 compared to the saline + D group.

The duration of drug/vehicle residence in the middle ear was evaluated with micro-CT (Figure 3). In the dual-vehicle + D group, the visible volume of the drug/vehicle was 49.52 ± 9.93 (relative value) at 0–1 h after IT drug delivery (Figure 3B). Within the first 5 days, the visible volume decreased rapidly to 10.92 ± 4.67. However, the decrease was very slow thereafter (from 10.92 ± 4.67 to 7.11 ± 2.83 over 44 days). In the saline + D group, the visible volume of the drug/vehicle was significantly smaller at 0–1 h after IT drug delivery (15.47 ± 9.13; *p* < 0.001) compared to the dual-vehicle + D group. Moreover, the drug/vehicle was not visible at 24 h or thereafter. The duration of drug/vehicle residence in the middle ear according to micro-CT was ≥48.1 ± 3.6 and 0.0 ± 0.0 days in the dual-vehicle + D and saline + D groups, respectively (Figure 3).

### Assessing the Hearing Threshold Based on the ABR

**FIGURE 4:**
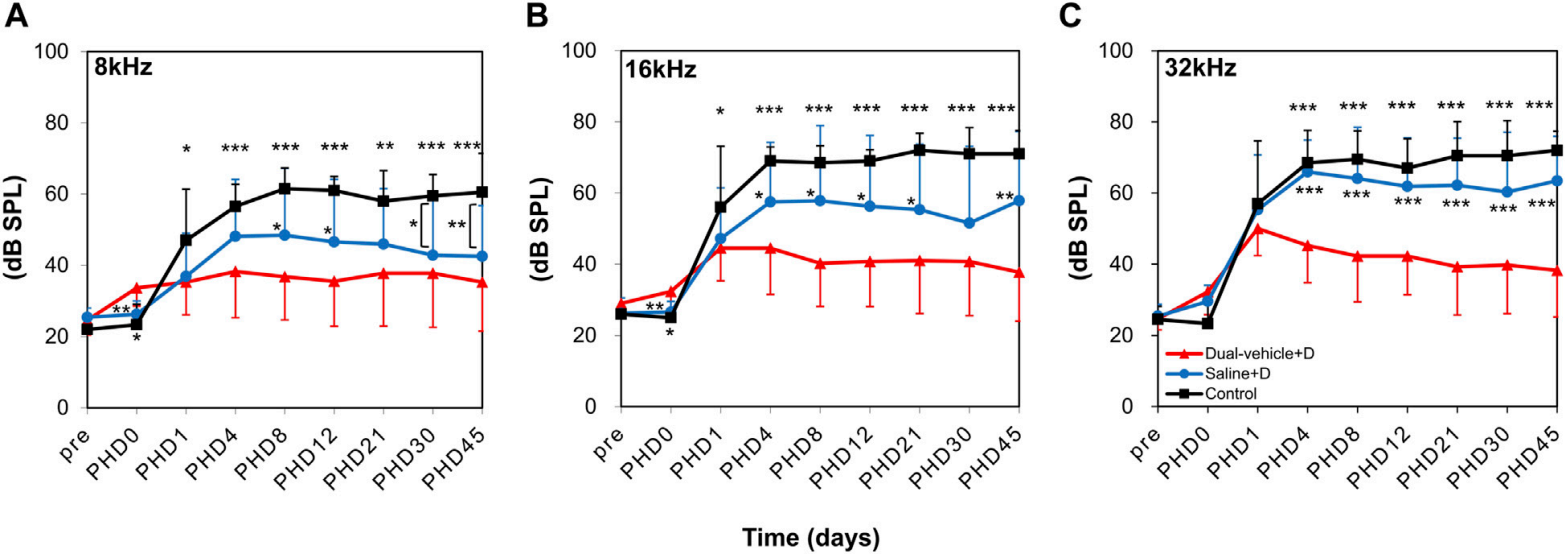
Changes in hearing thresholds. Auditory brainstem response thresholds were measured at **(A)** 8, **(B)** 16, and **(C)** 32 kHz on post-hearing loss day (PHD) 1, 4, 8, 12, 21, 30, and 45. Hearing was significantly better in the dual-vehicle + D group than in the saline + D or control group from PHD 4 to the final day of evaluation (PHD 45). *p < 0.05, **p < 0.01, ***p < 0.001 compared to the dual-vehicle + D group.

The hearing threshold was normal (<35 dB SPL at all three frequencies) in all animals before the experiment. At 1 day after the induction of ototoxic hearing loss (PHD 1), hearing deteriorated in all animals (Figure 4). Four days later, the hearing deterioration stopped in the dual-vehicle + D group but not in the saline + D or control groups. The hearing threshold was significantly better in the dual-vehicle + D group than in the saline + D group (*p* = 0.089 at 8 kHz, *p* = 0.012 at 16 kHz, and *p* < 0.001 at 32 kHz) or the control group (*p* < 0.001 at 8 kHz, *p* < 0.001 at 16 kHz, and *p* < 0.001 at 32 kHz) on PHD 4. Over the entire follow-up period (up to PHD 45), the hearing threshold in the dual-vehicle + D group was preserved or improved. On the contrary, the hearing threshold did not improve or deteriorated in the saline + D and control groups. The hearing threshold was always lowest in the dual-vehicle + D group versus the two other groups for all three frequencies (Figure 4). The final outcome was significantly better in the dual-vehicle + D group compared to the saline + D (*p* = 0.067 at 8 kHz, *p* = 0.001 at 16 kHz, and *p* < 0.001 at 32 kHz) and control (*p* < 0.001 at 8 kHz, *p* < 0.001 at 16 kHz, and *p* < 0.001 at 32 kHz) groups. The hearing threshold in the saline + D group was better than that in the control group at 8 kHz (*p* = 0.003). However, no significant differences were observed at 16 kHz (*p* = 0.165) or 32 kHz (*p* = 0.109).

### Organ of Corti Surface Preparation and Hair Cell Count

**FIGURE 5:**
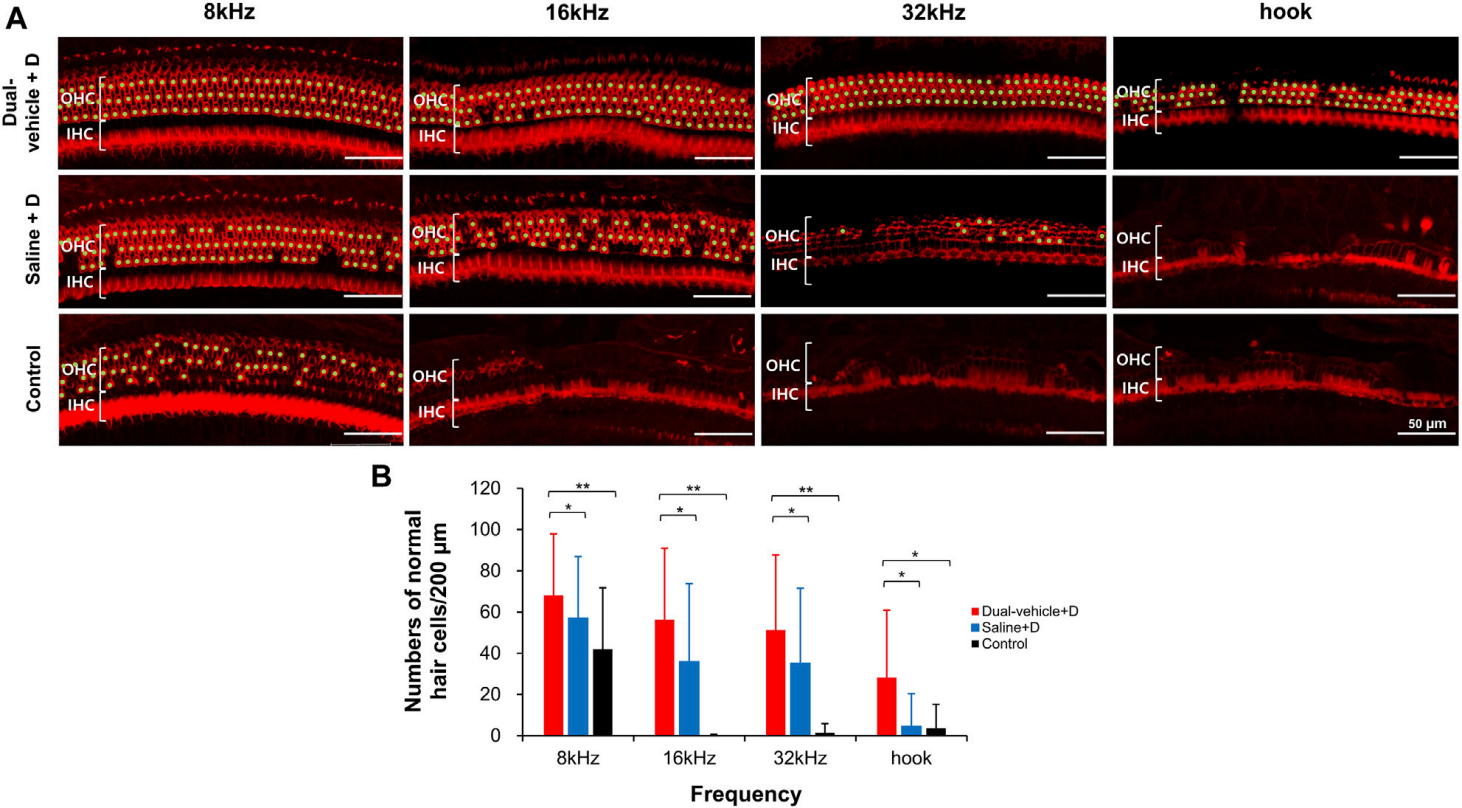
Number of outer hair cells in the apical turn (8 kHz), middle turn (16 kHz), basal turn (32 kHz), and hook portion. Significantly more outer hair cells were observed in the dual-vehicle + D group than in the saline + D or control group **(B)**. This finding was consistent along the entire length of the cochlea spiral. Live hair cells were marked with a light green dot **(A)**. *p < 0.05, **p < 0.01. IHC, inner hair cell; OHC, outer hair cell. Scale bars represent 50 µm.

The number of outer hair cells per 200 µm in the apical turn, middle turn, basal turn, and hook portion are shown in Figure 5. In the apical turn (relevant for 8-kHz hearing), significantly more live outer hair cells (*p* = 0.002) were observed in the dual-vehicle + D group (68.2 ± 29.7) than in the control group (42.0 ± 29.9). The saline + D group exhibited an intermediate outcome (57.1 ± 29.9), with a hair cell count that was significantly smaller (*p* = 0.021) than that in the dual-vehicle + D group, but greater than that in the control group. Similar trends were found for the middle turn (relevant for 16-kHz hearing) and basal turn (relevant for 32-kHz hearing). That is, significantly more live hair cells (*p* = 0.001 at 16 kHz, *p* = 0.002 at 32 kHz) were observed in the dual-vehicle + D group (56.4 ± 34.5 at 16 kHz, 51.3 ± 36.5 at 32 kHz) than in the control group (0.2 ± 0.5 at 16 kHz, 1.4 ± 4.4 at 32 kHz). Also, significantly more live hair cells (*p* = 0.039 at 16 kHz, *p* = 0.03 at 32 kHz) were observed in the dual-vehicle + D group than in the saline + D group (35.9 ± 37.9 at 16 kHz, 35.1 ± 36.4 at 32 kHz). In the hook portion, the dual-vehicle group exhibited a superior treatment outcome (28.2 ± 32.8) compared to the saline + D (4.6 ± 15.8, *p* = 0.023) and control (3.7 ± 11.5, *p* = 0.035) groups.

### Middle Ear Histology

**FIGURE 6:**
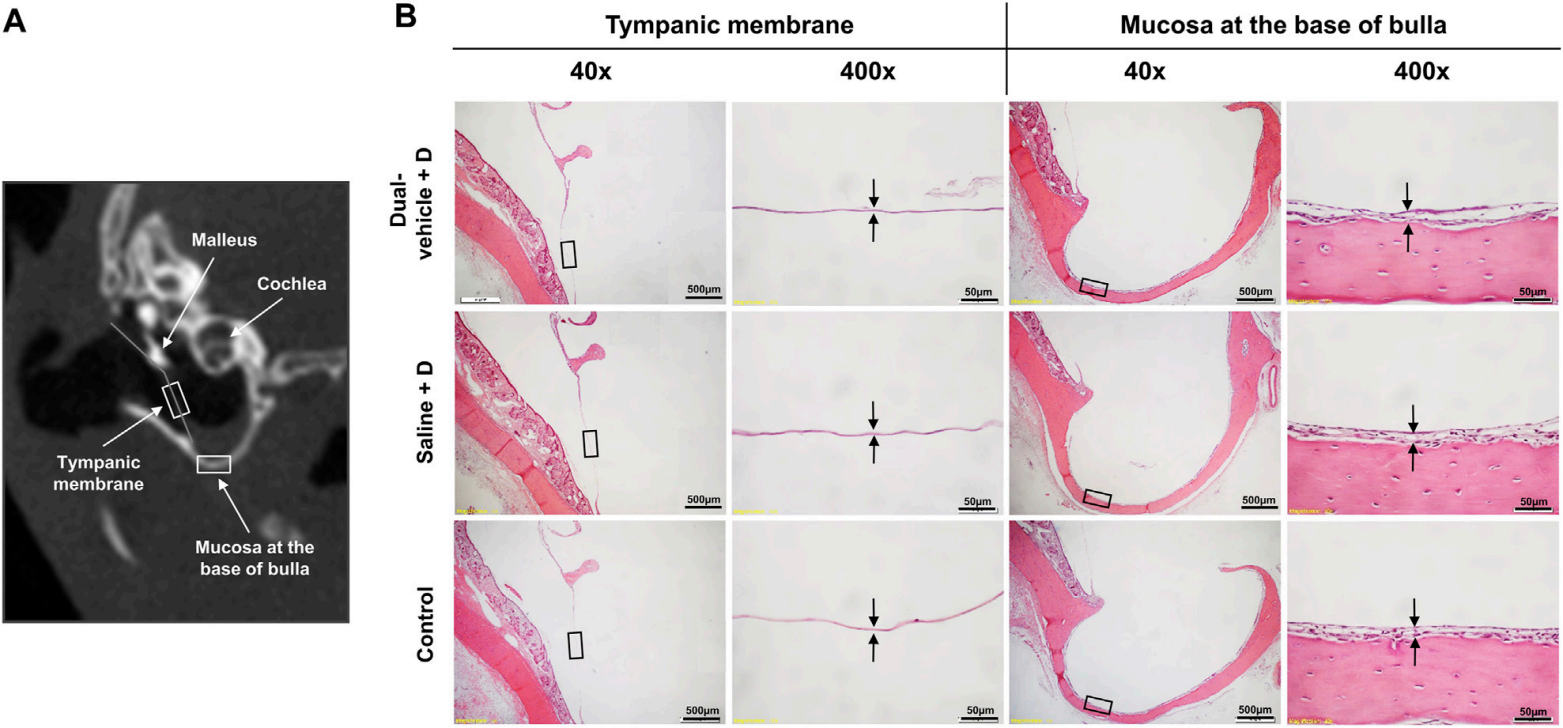
Histology of the tympanic membrane (TM) and mucosa at the base of the bulla (BB). **(A)** The two most vulnerable anatomic locations, the TM and BB, were evaluated histologically for evidence of inflammation or infection. **(B)** The samples were stained with hematoxylin and eosin. There were no differences in the qualitative morphology or quantitative thickness of the TM or BB mucosa among the three groups.

The mean thickness of the TM was 2.2 ± 0.6 μm in the dual-vehicle + D group, 1.9 ± 0.3 μm in the saline + D group, and 2.1 ± 1.0 μm in the control group, with no significant difference among the groups. The mean thickness of the BB mucosa was 16.1 ± 10.3 μm in the dual-vehicle + D group, 20.8 ± 11.6 μm in the saline + D group, and 13.7 ± 6.3 μm in the control group, with no significant difference in mucosal thickness among the groups (Figure 6).

## Discussion

The results from this study showed that ITD administration *via* the dual viscosity mixture vehicle is an effective and safe method for preventing ototoxic hearing loss. The hearing threshold was significantly better in the dual-vehicle + D group (35.3–38.3 dB SPL) compared to the saline + D group (42.5–63.4 dB SPL) and control group (60.5–72.0 dB SPL). Morphologic evaluation of the cochlear hair cells also supported this finding. There were significantly more hair cells in the dual-vehicle + D group (28.2–68.2) than in the saline + D (4.6–57.1) or control (0.2–42.0) group. Clinically there is no effective method to prevent drug induced hearing loss (ototoxic hearing loss) in patients undergoing chemotherapy and anti-tuberculosis therapy. Also, the dual viscosity mixture vehicle was highly biocompatible. That is, there was no ear with inflammation, infection, or permanent TM perforation (incidence of adverse effect was 0%), as indicated in the TM endoscopy, micro-CT, and middle ear histology results. It is encouraging that the histology of the TM and middle ear mucosa in the dual-vehicle + D group was identical to that of the control group that did not receive any intervention. These results are in line with previous studies that had confirmed the safety of HA as an IT drug delivery vehicle (Borden et al., 2011; Park et al., 2020). Considering that dexamethasone mixed in saline is currently the standard-of-care strategy for ITD administration, the dual viscosity mixture vehicle may be a potentially superior alternative for IT drug delivery.

It is not clear why the treatment outcome was best in the dual-vehicle + D group; however, there are several theories. First, the long-lasting drug/vehicle in the middle ear might have served as a potent drug depot. Due to the high viscosity and adhesive property (Supplementary video S1) of the outer vehicle (El Kechai et al., 2016), it is less likely for the dual vehicle to drain though the Eustachian tube compared to saline. Our micro-CT data showed that the dual vehicle stayed in the middle ear for several weeks. This residual volume of drug/vehicle can provide a continuous drug supply to the inner ear (Hwang et al., 2021). When conventional HA was used as a vehicle, the vehicle stayed in the middle ear for only 2 days and the treatment effect was limited (Park et al., 2020). But this hypothesis is a speculation and needs further verification. Second, the timing of ITD administration might have been ideal for the dual-vehicle + D group. The biological effect of encapsulated dexamethasone is presumed to reach its peak after several days (El Kechai et al., 2016). Given that ototoxic hearing loss developed on the third and fourth day after ITD administration, this might have coincided perfectly with the timing of peak preventative and therapeutic effects in the dual-vehicle + D group. Meanwhile, the optimal time for attaining peak effects in the saline + D group might have been different (probably shorter). Also, because the drug/vehicle only lasted for <24 h, some preventative effects might have been exerted in the saline + D group, but definitely no therapeutic effects, after ototoxic insult. Third, the high motorization property of HA might have facilitated the diffusion of dexamethasone through the RW. Dexamethasone can penetrate through the inner ear by diffusion and reach the perilymph successfully (Himeno et al., 2002; Plontke and Salt, 2003; Salt, 2008). By increasing the permeability of the RW for a prolonged duration, it might have been easier for dexamethasone to penetrate and diffuse across the RW membrane. Others have also reported that HA can enhance the permeability of the RW (Selivanova et al., 2003). Fourth, the dual vehicle might have prevented the initial burst release of dexamethasone and prolonged the release time. Given that dexamethasone was mixed inside a multiphase vehicle, it would take a longer time for it to be released fully. Multivesicular vehicles such as liposome also provide a strong sustained release (Kim et al., 1983). Whereas it took only 1 day for saline to release 80% of the dexamethasone, it took 7 days for a multivesicular liposome gel to release the same amount (Li et al., 2020).

Compared to the control group, the final hearing benefit in the dual-vehicle group was approximately 25–35 dB, which is significant from a clinical point of view. The hearing benefit was only 10–15 dB in a previous study that used single-phase HA as the vehicle (Park et al., 2020); this small effect might be attributable to the low molecular weight of HA used in the study. In another study that investigated the effect of HA gel with liposomes loaded with dexamethasone, there was no noticeable hearing recovery (Mamelle et al., 2018). The concept of liposome as a vehicle is similar to that of our inner low-molecular-weight HA microsphere encapsulation. However, it seems that liposome is not as effective as the dual viscosity mixture of HA. There have been reports describing the use of dexamethasone-loaded, chitosan-based, genipin-cross-linked hydrogel delivery systems. According to the results, the hearing benefit was approximately 15–25 dB (Yüksel Aslıer, 2019). Although our dual vehicle was not able to completely cure the ototoxic hearing loss, the hearing outcome was noticeably improved compared with those of previous reports.

The treatment effect was most pronounced at a high frequency (32 kHz), which can be explained by the tonotopic arrangement of the cochlear hair cells. When the drug is delivered *via* an IT route, the port of entry is the RW and/or OW. This will generate a gradient in drug concentration that is higher in the basal turn and lower in the apical turn (Plontke et al., 2008). Due to a dose–response relationship, the hearing gain should be better at a higher frequency compared to a lower frequency. The good treatment effect in the high-frequency region was also reflected by the hair cell count. Although the absolute number of hair cells was greater in the apical turn, the difference between the dual-vehicle group and control group was more evident in the mid-to-basal turn. Others have also reported a greater contrast in hair cell counts (Li et al., 2020) or preservation of stereociliary structures in the basal turn (Yüksel Aslıer, 2019) after IT drug delivery. The tonotopic gradient can also be demonstrated by IT neomycin (an ototoxic drug) administration. When neomycin-loaded HA was administered in the middle ear, hair cell loss was most evident in the basal turn, whereas the apical turn was relatively preserved (Saber et al., 2009). It seems that drugs that enter the inner ear diffuse slowly along the perilymphatic chamber from the base to the apex (Salt and Ma, 2001).

There were several limitations in this study. First, we did not perform a pharmacokinetic study to measure the drug concentration inside the cochlea. To fully understand why the treatment outcome is superior in the dual-vehicle group, we must investigate the concentration of dexamethasone at several time points. We are planning on a high-performance liquid chromatography study to clarify this point in the near future. Second, varying the timing of ITD administration may help elucidate the optimal treatment time point. Finding this optimal time point is clinically important, because it differs depending on the vehicle. Third, histologic evaluation of the TM and middle ear mucosa was only performed at one time point (at the end of the study). If transient inflammation was present during the early phase, we might have missed this finding. However, we believe this point does not greatly undermine safety, as we evaluated the TM *via* endoscopy throughout the experiment; additionally, transient and subtle events with no sequelae are not clinically critical. Fourth, micro-CT cannot differentiate between the drug and the vehicle. In future studies, contrast agents or fluorescent dyes that can bind to the drug chemically would be helpful; however, to our knowledge, there is no currently applicable CT contrast agent for this purpose. Fifth, we did not perform a nuclear stain for the cytocochleogram. This would have helped us understand the impact of dual vehicle on the nuclei of the hair cells and verify the cell count. Sixth, the long-term stability of the dual phase colloidal system should be verified. Seventh, the causal relationship between high viscosity and a better treatment outcome has not been proven in this study. Eighth, we did not provide an *in vitro* release study of dexamethasone from this vehicle in a simulated low volume environment to show it’s release characteristic and mechanism of release.

## Data Availability

The original contributions presented in the study are included in the article/Supplementary Material, further inquiries can be directed to the corresponding authors.
